# Tunable Topological Energy Bands in 2D Dialkali‐Metal Monoxides

**DOI:** 10.1002/advs.201901939

**Published:** 2020-01-07

**Authors:** Chenqiang Hua, Si Li, Zhu‐An Xu, Yi Zheng, Shengyuan A. Yang, Yunhao Lu

**Affiliations:** ^1^ Zhejiang Province Key Laboratory of Quantum Technology and Device and Department of Physics in Zhejiang University State Key Lab of Silicon Materials School of Materials Science and Engineering in Zhejiang University Hangzhou 310027 P. R. China; ^2^ School of Physics and Electronics Hunan Normal University Changsha Hunan 410081 China; ^3^ Research Laboratory for Quantum Materials in Singapore University of Technology and Design Singapore 487372 Singapore; ^4^ Center for Quantum Transport and Thermal Energy Science School of Physics and Technology in Nanjing Normal University Nanjing 210023 China; ^5^ Zhejiang Province Key Laboratory of Quantum Technology and Device and Department of Physics in Zhejiang University Hangzhou 310027 P. R. China; ^6^ Collaborative Innovation Centre of Advanced Microstructures in Nanjing University Nanjing 210093 P. R. China

**Keywords:** 2D topological states, dialkali‐metal monoxides, emergent fermions, strain engineering, topological phase transition

## Abstract

2D materials with nontrivial energy bands are highly desirable for exploring various topological phases of matter, as low dimensionality opens unprecedented opportunities for manipulating the quantum states. Here, it is reported that monolayer (ML) dialkali‐metal monoxides, in the well‐known 2H‐MoS_2_ type lattice, host multiple symmetry‐protected topological phases with emergent fermions, which can be effectively tuned by strain engineering. Based on first‐principles calculations, it is found that in the equilibrium state, ML Na_2_O is a 2D double Weyl semimetal, while ML K_2_O is a 2D pseudospin‐1 metal. These exotic topological states exhibit a range of fascinating effects, including universal optical absorbance, super Klein tunneling, and super collimation effect. By introducing biaxial or uniaxial strain, a series of quantum phase transitions between 2D double Weyl semimetal, 2D Dirac semimetal, 2D pseudospin‐1 metal, and semiconductor phases can be realized. The results suggest monolayer dialkali‐metal monoxides as a promising platform to explore fascinating physical phenomena associated with novel 2D emergent fermions.

## Graphical abstract

2H‐MoS_2_ type metal‐shrouded oxides named dialkali‐metal monoxides are predicted to host multiple topological phases with exotic quasiparticle excitations, including double Weyl, 2D Dirac, 2D pseudospin‐1 metal, and semiconductor phases. These phases can be effectively tuned and manipulated by strain engineering and can be manifested by various fascinating effects such as super Klein tunneling and super collimation effect.

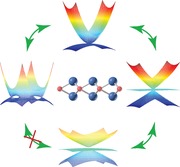

## 1 Introduction

The flourishing of 2D materials and the technical feasibility of fabricating astonishingly complex van der Waals heterostructures^1^ allow extremely rich quantum phases of matter to be explored and tuned in atomic thin‐films with single‐crystal quality. Following the ground‐breaking experiments on graphene,^2^, ^3^, ^4^ a variety of archetypal 2D materials have been reported, such as group‐(III to VI) elemental 2D layers,^5^, ^6^, ^7^, ^8^ transition‐metal dichalcogenides (TMDCs),^9^, ^10^, ^11^, ^12^ black phosphorous^13^, ^14^, ^15^ and its binary main group counterparts,^16^, ^17^, ^18^ InSe family materials,^19^ and 2D MXenes.^20^, ^21^ Very recently, various 2D materials with ferromagnetic and/or ferroelectric ordering have also been explored.^22^, ^23^, ^24^, ^25^, ^26^, ^27^, ^28^

In this field, the search for 2D materials with nontrivial topological band structures is an intriguing research topic. It is motivated by recognizing that many unusual properties of graphene are connected to its special topological band structure: the conduction and valence bands linearly cross at symmetry‐protected nodal points, such that the low‐energy quasiparticles behave like Dirac fermions with zero effective mass. Currently, a target in this direction is to explore new types of band degeneracy points and the associated new emergent fermions. For example, previous works have theoretically proposed 2D massive Dirac fermions,^29^ double Weyl fermions, and pseudospin‐1 fermions in some 2D materials, such as blue phosphorene oxide^30^ and monolayer (ML) Mg_2_C.^31^ However, these proposed materials are not ideal because the emergent fermions are not realized in their equilibrium states. They require finite externally applied strains to be tuned into double Weyl semimetal or the pseudospin‐1 metal states, which is challenging to control in experiment. Thus, it still remains as a challenge to search for suitable 2D materials which can host these new fermions.

Meanwhile, among the 2D materials family, the metal‐shrouded MXenes have been attracting great interest due to their rich properties.^20^, ^32^, ^33^ Most members of the family are carbides and nitrides (e.g., Ti_2_C, Ti_2_N, and Nb_2_C),^33^, ^34^ consisting of three atomic layers in the order of *M–X–M*, where *M* is the transition metal and *X* is C or N. Recently, the study has been extended to metal‐shrouded oxides.^35^ For example, the 2D Tl_2_O with the same structure has been proposed. Considering that Tl has properties similar to alkali metal elements, it is natural to ask whether alkali metal oxides (by replacing Tl with alkali metal) can also be stabilized in a 2D crystal form? If so, this may open the door to a new family of 2D materials, i.e., 2D metal‐shrouded oxides. In view of the great variety of 2D metal‐shrouded MXenes and their rich properties, one can expect that 2D metal‐shrouded oxides will also grow into a big family with fascinating properties.

**Figure 1:**
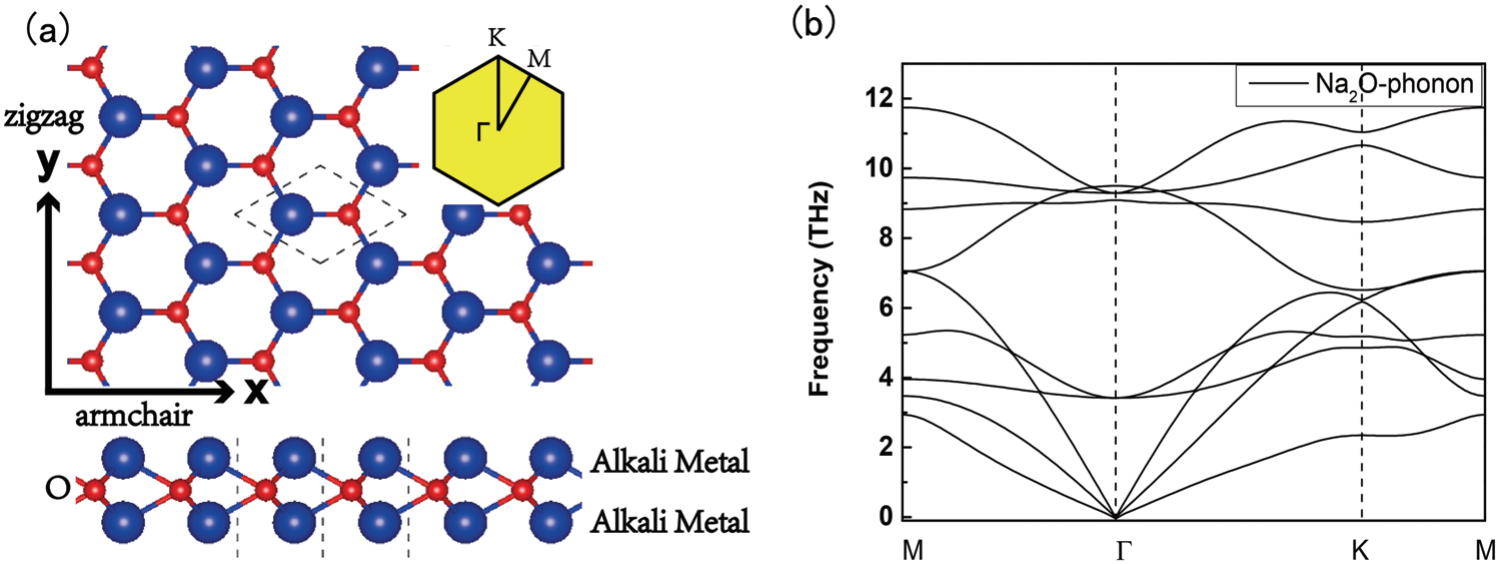
a) Top and side view of monolayer dialkali‐metal monoxides. Inset is the first Brillouin zone with high‐symmetry points. b) Phonon spectrum of ML Na_2_O, showing no imaginary mode in the whole Brillouin zone.

In this work, we try to address the above challenge and question by proposing a new family of 2D material—monolayer dialkali‐metal monoxides (DMMOs) with the 2H‐MoS_2_ structure. Based on first‐principles calculations, we show that Na_2_O and K_2_O can be stabilized in this 2D lattice as shown in **Figure** **1**a. They exhibit good stability and mechanical property. Most importantly, these materials host a diverse of 2D topological phases with new emergent fermions. In the equilibrium state, ML Na_2_O is a 2D double Weyl (massive Dirac) semimetal, while ML K_2_O is a nearly ideal 2D pseudospin‐1 metal. To our best knowledge, they represent the first examples that host the 2D double Weyl semimetal and the pseudospin‐1 metal in the equilibrium state. Furthermore, the topological phase and the novel fermions can be effectively controlled by strain. Under biaxial strain, a series of topological phase transitions between 2D Dirac metal, double Weyl semimetal, pseudospin‐1 metal, and semiconductor phases can be realized. Particularly, when uniaxial strain is applied, an intriguing transition from double Weyl point to two single Weyl (Dirac) points is triggered, which would be manifested in quantum transport measurements by a drastic changeover from weak localization to weak antilocalization. This kind of phase transition has not been reported for realistic materials before. In addition, these emergent fermions will lead to a range of exotic effects, including universal optical absorbance, super Klein tunneling, and super collimation effect, which have been predicted but not experimentally demonstrated yet due to the lack of suitable materials. Thus, our discovery not only predicts a family of new 2D materials but also provides an experimentally feasible platform to explore new emergent fermions and their fascinating fundamental physical effects.

## 2 Results and Discussions

The proposed ML dialkali‐metal monoxides A_2_O (A = Na, K) take the same structure as ML 2H‐MoS_2_, in which three atomic layers are stacked in the A–O–A sequence with the D_3h_ point group symmetry, forming the *P* $\bar{6}$ *m*2 space group (No. 187). As depicted in Figure 1, the top view of DMMO lattice is honeycomb like, corresponding to a hexagonal first Brillouin zone (BZ). The optimized lattice parameters for Na_2_O and K_2_O are 3.49 and 3.86 Å, respectively.

We calculated the phonon spectra of DMMOs to investigate the dynamical stability of these compounds. The result for ML Na_2_O is shown in Figure 1b. Apparently, there is no imaginary mode in the whole Brillouin zone, indicating that the material is dynamically stable. The phonon spectrum of K_2_O is similar to Na_2_O, as shown in the Supporting Information. Excellent energetic stability has also been validated, as evident by the large cohesive energy of −8.02 and −7.36 eV per formula (f.u.^−1^) for Na_2_O and K_2_O, respectively. These results suggest the experimental feasibility to synthesize the proposed monolayer materials. For the other DMMOs (*A* = Li, Rb, Cs), we find that Li_2_O has some imaginary phonon modes while Rb_2_O, and Cs_2_O is extremely unstable. The calculation results (including their band structures) are summarized in the Supporting Information.

**Figure 2:**
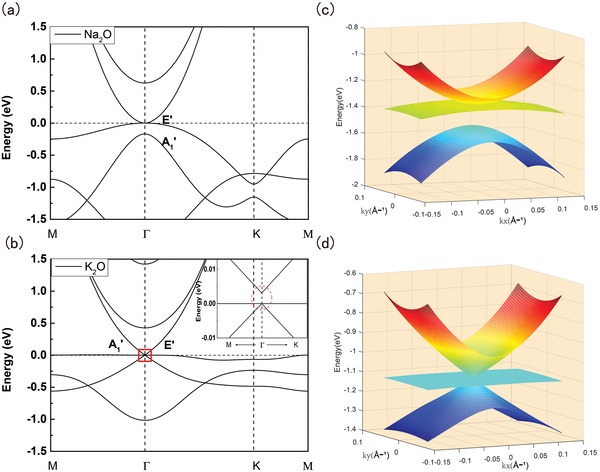
a,b) Band structures of ML Na_2_O and ML K_2_O, respectively. *E*′ and *A*_1_′ are the irreducible representations of the D_3h_ point group, which contribute three bands near Fermi level. The inset of (b) is the zoom‐in of the energy bands of K_2_O at *E*_F_ near the Γ point. c,d) 2D energy dispersion near the Fermi surface, which are massive Dirac point and triply degenerate pseudospin‐1 point for ML Na_2_O and K_2_O, respectively.

We now focus on the electronic structures of the monolayer DMMOs. In **Figure** **2**, we plot the calculated band structures for ML Na_2_O and K_2_O. The band structures of Li_2_O are similar to K_2_O, as summarized in Figure S8 in the Supporting Information. It is noteworthy that, due to the small atomic numbers, spin–orbit coupling (SOC) has negligible effect on the band structure of DMMOs. Consequently, electron spin can be regarded as a dummy degree of freedom and the DMMOs can be treated as effective spinless systems. In the following, unless explicitly stated, we will not take spin degeneracy into account when analyzing the band topology. As shown in Figure 2, both materials are metallic without a bandgap, due to the presence of three energy bands near the Fermi level, originating from the *E*′ and *A*_1_′ irreducible representations of the D_3h_ point group (see Figure 2). For Na_2_O, the quadratic conduction and valence bands touch at a single degenerate point at the high symmetry Γ point (Figure 2c). Without external charge doping, such point also defines the Fermi energy due to ionic band filling. The band dispersion around this point is quadratic in all directions in the 2D plane, so this point is named as a 2D double Weyl point similar to that in blue phosphorene oxide and monolayer Mg_2_C. However, unlike blue phosphorene oxide and Mg_2_C which both require applied strain to realize such double Weyl point, ML Na_2_O is intrinsically a 2D double Weyl semimetal. On the other hand, for ML K_2_O, the three *E*′ and *A*_1_′ bands cross each other at the Γ point, forming a distinctive triply degenerate point Fermi surface (see the Supporting Information). The difference is mainly due to stronger ionic bonding in ML K_2_O. Consequently, in the vicinity of triply degenerate point, the upper and lower bands are characterized by graphene‐like linear dispersion, intersected by a very flat band at the Fermi point (Figure 2d). Such unique triply degenerate point Fermi surface in ML K_2_O corresponds to pseudospin‐1 fermions, as we will elaborate in the flowing paragraphs.

The triple band degeneracy point and the associated pseudospin‐1 fermions can be well modelled by the ***k***·***p*** method, taking the DMMO lattice symmetry constraints into account. Since the band degeneracy points are located at the Γ point, we construct the effective model using the *E*′ and *A*_1_′ states as bases. Subjected to time reversal symmetry and D_3h_ symmetry operations, which include threefold rotation C_3_, twofold rotation *C*_2_, and horizontal mirror *M*_h_, the ***k***·***p*** Hamiltonian expanded up to *k*‐quadratic order have the general form of

(1) $$H_{0}\left(k\right)=\left[\begin{array}{ccc} M_{1}+B_{3}k^{2} & C\left(k_{y}^{2}-k_{x}^{2}\right)-iAk_{x} & 2Ck_{x}k_{y}-iAk_{y}\\ C\left(k_{y}^{2}-k_{x}^{2}\right)+iAk_{x} & M_{2}+B_{1}k_{x}^{2}+B_{2}k_{y}^{2} & \left(B_{1}-B_{2}\right)k_{x}k_{y}\\ 2Ck_{x}k_{y}+iAk_{y} & \left(B_{1}-B_{2}\right)k_{x}k_{y} & M_{2}+B_{2}k_{x}^{2}+B_{1}k_{y}^{2} \end{array}\right]$$

where *k* = |***k***| is the magnitude of 2D wave vector, *M*_1,2_, *A*, *B*_1,2,3_, and *C* are band parameters which are extracted by fitting the density functional theory (DFT) band structures. Exactly at Γ point (*k* = 0), one can see that *M*_1_ and *M*_2_ represent the energies of the *A*_1_′ and the *E*′ states, respectively. By determining the band parameters of this general Hamiltonian, we can quantitatively understand the pronounced changes in the low‐energy band structures of Na_2_O and K_2_O.

First, for Na_2_O, *E*′ has higher energy than *A*_1_′, meaning *M*_1_ is smaller than *M*_2_. With a unitary transformation, we can project the *H*_0_ (***k***) model onto the two *E*′ bands around the double Weyl (massive Dirac) point. The resulting two‐band model is written by

(2) $$H_{\mathrm{DW}}\left(k\right)=B_{+}\left(k_{y}^{2}+k_{x}^{2}\right)+\left[\begin{array}{cc} 0 & B_{-}k_{-}{}^{2}\\ B_{-}k_{+}{}^{2} & 0 \end{array}\right]$$

where *B*_±_ = 0.5(*B*_2_ ± *B*_1_), *k*_±_ = *k_x_* ± *ik_y_*, and the energy is measured from the degenerate point. Without the diagonal term, the model *H*_DW_ resembles that of the AB‐stacked bilayer graphene, which features a Berry phase of 2π, doubling that of a single Dirac point as in monolayer graphene.^3^ However, for bilayer graphene, there are two such points at BZ corners related by the time reversal symmetry; whereas for ML Na_2_O, there is only one double Weyl point sitting at the BZ center. The double Weyl point endows ML Na_2_O with an interesting optical property, i.e., it should exhibit a universal optical absorbance of *πα* ≈ 2.3% (α is fine‐structure constant) at low frequencies [for ℏω < (*M*_2_ – *M*_1_) here].^30^, ^36^

For K_2_O, the triply degenerate point can be explained by setting *M*_2_ = *M*_1_ = 0 (by neglecting the insignificant gap). In this case, the *k*‐linear terms dominate the low energy spectrum, which allows us to further simplify the Hamiltonian *H*_0_ into the following form

(3) $$H_{PS1}\;\left(k\right)=\left[\begin{array}{ccc} 0 & -iAk_{x} & -iAk_{y}\\ iAk_{x} & 0 & 0\\ iAk_{y} & 0 & 0 \end{array}\right]=Ak\cdot S$$

where ***S*** is pseudospin‐1 angular momentum vector.^37^ Although *H*_*PS*1_ mimics the Hamiltonian of 3D Weyl, each ***S*** matrix is 3 × 3, representing the triply degeneracy of pseudospin‐1 fermions in K_2_O near the Fermi surface. In contrast to blue phosphorene oxide and Mg_2_C, the middle intersecting band is extremely flat, nearly extending over the whole Brillouin zone. Intriguingly, ML K_2_O exhibits pseudospin‐1 phase in the equilibrium state, which is critical for experimental exploration of exotic quantum effects associated with pseudospin‐1 fermions, such as super Klein tunneling with almost 100% transmission probability for large incident angles,^37^, ^38^ and super collimation, which means guided unidirectional transport in the presence of a periodic potential, regardless of its initial direction of motion. Monolayer K_2_O may provide a promising platform for observing these fascinating phenomena.

It is well known that the physical properties of 2D materials can be effectively tuned by strain, like the bandgap engineering of 2H‐TMDC semiconductors.^39^ Strain has also been proposed to effectively modulate dielectric properties,^40^ spin–orbit coupling,^41^ thermal conductivity^42^ and interlayer coupling (and mismatch) in vdW heterostructures^43^ in 2D crystals. In the following, we will show that strain can induce interesting multiple topological phase transitions in the ML DMMOs. Before that, we introduce the in‐plane stiffness constant, defined as $C\;=\frac{1}{S_{0}}\;\frac{\partial^{2}E_{s}}{\partial \varepsilon^{2}}$, where *S*_0_ represents the area of the unstrained cell and *E*_S_ is the energy difference between unstrained and strained systems. Our calculation shows that the stiffness constants for Na_2_O and K_2_O are about 57.4 and 38.9 N m^−1^, respectively. These values are significantly smaller than archetypal 2D systems of graphene (≈340 N m^−1^)^44^ and MoS_2_ (≈180 N m^−1^),^45^ indicating that the properties of ML DMMOs can be readily manipulated by external strain. Strain stress curves are depicted in Figure S4 in the Supporting Information, which gives a maximum stress of about 2 GPa within ±10% biaxial strain. If taking the effective thickness as 2.85 Å, the instant maximum stress is 0.57 N m^−1^.

**Figure 3:**
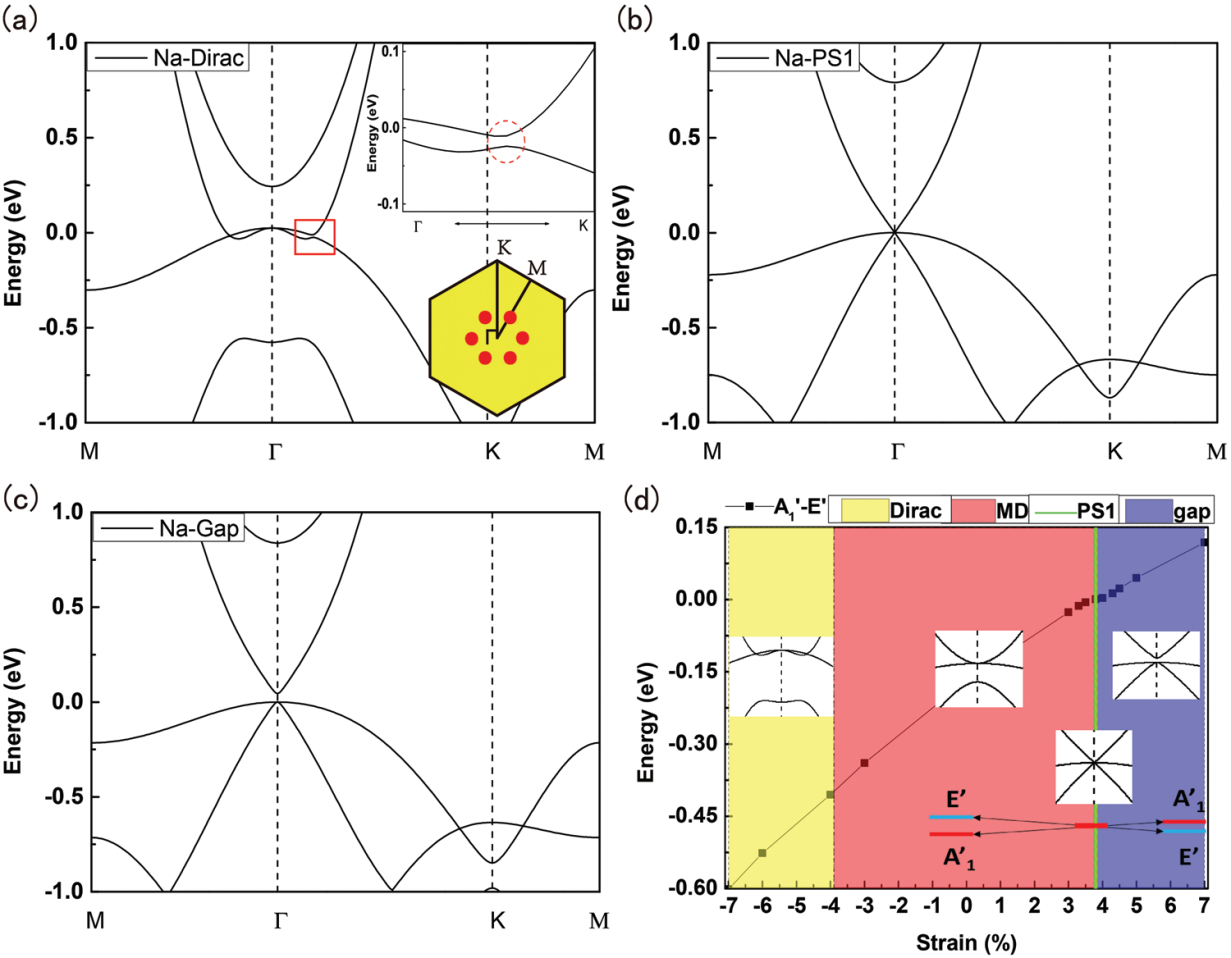
Band structures of ML Na_2_O under biaxial strain of a) −7%, b) 3.8%, and c) 5%. Inset of (a) shows the emergence of six symmetry protected single Dirac points (red dots) along Γ‐M paths. d) The complete strain versus topological phase diagram of ML Na_2_O. The solid line represents the energy difference between the *A*_1_′ and *E*′ representations. Four topological quantum states can be reversibly tuned by biaxial strain in ML Na_2_O.

We first show the changes in electronic band structure by applying biaxial strain. For Na_2_O, multiple quantum phases can be generated, as shown in **Figure** **3**. With a large compressive strain (for example −7% in Figure 3a), the double Weyl semimetal of Na_2_O becomes metallic with six extra Dirac points (due to historical reasons, spin–orbit‐free Weyl points in 2D are also referred to as Dirac points^46^) emerging along the Γ‐M axes apart from the original point at Γ. These new Dirac points are protected by three vertical mirror planes, while the band crossing along Γ‐K is gapped without symmetry protection. When applying tensile strain, the energy difference between *E*′ and *A*_1_′ decreases, leading to accidental band touching at Γ between three bands at ≈3.8% strain. After this topological phase transition, Na_2_O becomes a pseudospin‐1 metal like pristine ML K_2_O. Further increase in tensile strain causes band inversion between *E*′ and *A*_1_′, and drives the system into a semiconductor, as shown in Figure 3c. We have plotted the complete quantum phase versus strain diagram of Na_2_O in Figure 3d. The results for ML K_2_O under biaxial strain are similar to Na_2_O, which is summarized in the Supporting Information.

**Figure 4:**
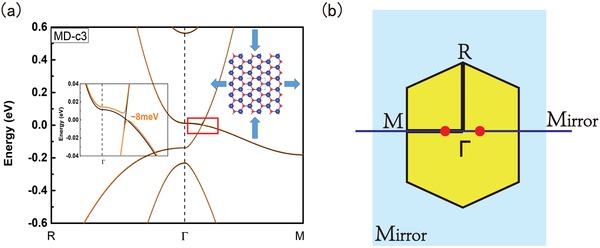
a) Band structure of ML Na_2_O under uniaxial‐strain of −3% applied along the mirror plane. When SOC is included, a nontrivial gap of ≈8 meV is determined (the orange curve). b) Schematic of the location of symmetry protected massless Dirac points (red dots) induced by uniaxial strain.

Next, we consider the effects of uniaxial strain, which has been experimentally employed in modifying the physical properties of graphene and TMDCs.^47^, ^48^, ^49^ Unlike biaxial strain, uniaxial strain changes the crystalline symmetry of DMMOs by explicitly breaking the threefold rotation symmetry and vertical mirror symmetry. For uniaxial strain applied along the mirror plane, the D_3h_ symmetry of DMMOs is reduced to C_2v_. The uniaxial strain effect on band structures is pronounced for the double Weyl semimetal phase in Na_2_O, as shown in **Figure** **4**. It is distinctive that due to the symmetry reduction, the original double Weyl point (corresponding to the *E*′ doublet) is destroyed. Instead, a new pair of linearly dispersed Dirac points, as required by time reversal symmetry, emerge along Γ‐M (see Figure 4a). Such a phase transition is consistent with the physical picture that a double Weyl point can be regarded as a superimposition of two single Weyl (Dirac) points, such as the case of bilayer graphene. To describe this strain‐induced topological phase transition, we incorporate the effect of such uniaxial strain in the original Hamiltonian *H*_DW_ by a perturbation term *H*_s_ constrained by the remaining symmetry, namely

(4) $$H\;\left(k\right)=H_{\mathrm{DW}}\;\left(k\right)+H_{s}$$

with

(5) $$H_{s}=-D\sigma_{x}$$

where we only keep the leading order term in *H*_s_. Indeed, the double Weyl (massive Dirac) point at Γ is splitting into two single Dirac points located at $k_{D}^{\pm }=(\pm \sqrt{D/B_{-}},0)$ on the *k_x_* axis (i.e., Γ‐M). Expanding the model at $k_{D}^{\pm }$ leads to 2D Dirac model

(6) $$H_{D}\;\left(q\right)=\;\pm \left(v_{+}q_{x}\sigma_{0}+v_{-}q_{x}\sigma_{x}+v_{-}q_{y}\sigma_{y}\right)$$

where the wave vector ***q*** is measured from $k_{D}^{\pm }$, ± corresponds to the two Dirac points, and $v_{\pm }=2B_{\pm }\;\sqrt{D/B_{-}}$. As we already mentioned above, the double Weyl point is characterized by a 2π Berry phase, whereas a single Dirac point corresponds to a π geometric phase. At low‐temperature, a 2π Berry phase is manifested by weak localization in quantum transport, whereas a π Berry phase leads to weak antilocalization. Thus, the uniaxial strain can induce an interesting transition from weak localization to weak antilocalization in ML DMMOs, which would be detectable by charge transport measurements. Due to the reduced symmetry, the aforementioned Dirac model also has a finite energy tilt term of ±*v*_+_ *q_x_σ*_0_, which may induce interesting squeezing effects on the Laudau level spectrum in magnetic field.^50^ It is noteworthy that, in this phase, we will get a 2D topological insulator state when SOC is included, as also shown by the orange curve in Figure 4a. Although the energy gap is not significant (≈8 meV), we find that the corresponding topological Z_2_ index is one, indicating a nontrivial 2D topological insulator (TI) state. The resulting topological edge state is plotted in Figure S5 in the Supporting Information, which also shows the evolution of Wannier charge center by means of WannierTools.^51^ By constructing certain vdW heterostructures, we should be able to further enhance the SOC gap in Na_2_O ML, making ML DMMOs promising 2D TI candidates for studying quantum spin Hall states.

It is well known that TMDCs exhibits 1T or 2H phase under different environment. Similarly, ML DMMOs also have 1T phase counterparts which are dynamically stable.^52^ For example, 1T‐Na_2_O could grow during the oxidation of Na (110) surface^53^ and 1T‐Cs_2_O may also be exfoliated from bulk.^54^ Comparing to 1T‐DMMOs, 2H‐DMMOs are metastable phase, e.g., 2H‐K_2_O is ≈0.45 eV higher per unit in energy than 1T‐K_2_O (≈0.65 eV for Na_2_O). However, these energy differences are much smaller than the energy difference between 1T and 2H phases of ML MoS_2_ (≈0.85 eV). Since the metastable 1T phase of ML MoS_2_ (not dynamically stable in theory^55^) has been already obtained in experiment^56^ and the phase transition between 1T‐MoS_2_ and 2H‐MoS_2_ can be controlled by charge doping,^57^ it is expected that 2H‐DMMOs can also be obtained in experiments (see Figure S6, Supporting Information). We also suggest that the proposed two exemplary 2D materials of ML Na_2_O and ML K_2_O are likely to be synthesized by molecular beam epitaxy (MBE) or by chemical vapor deposition method. Another possible method is using alkali thin substrate with adsorption of oxygen, similar to the surface selenization to get reversible transition between 2H and 1T PtSe_2_.^58^ Although alkali compounds are not stable by reacting with ambient moisture when exposed to the air environment, we can encapsulate DMMO monolayers by protection layers of chalcogen elements, nonreactive oxides, or 2D insulating materials (such as boron nitride (BN)) in the glove‐box environment before the device fabrication process. In situ synthesis and characterization techniques are also readily available and dialkali‐metal monoxides can be grown in ultrahigh vacuum environment by MBE method, probed subsequently by scanning tunnel microscope and angle‐resolved photoemission spectroscopy (ARPES) to determine the physical properties of these materials. For tuning topological states, strain can be introduced in ML DMMOs by transferring the thin films to flexible substrates or by using piezoelectric substrates. The induced topological phase transitions can be probed by scanning probe spectroscopy, ARPES, and ultimately by device fabrications and charge transport measurements.

## 3 Conclusions

In conclusion, we propose a family of new 2D materials—the monolayer dialkali‐metal monoxides with the 2H‐TMDC structure. These materials enjoy good stability and excellent flexibility. Most importantly, we find that these materials are novel types of 2D topological metals. In the equilibrium state, Na_2_O is a 2D double Weyl semimetal, and K_2_O is a 2D pseudospin‐1 metal. They may host a range of fascinating physical effects such as universal optical absorbance, super Klein tunneling, and super collimation effect. Furthermore, rich topological phase transitions can be achieved in these materials by strain. Under biaxial strain, a series of transitions can be realized. Under uniaxial strain, the double Weyl point can be split into a pair of single Weyl (Dirac) points, accompanied with the transition from weak localization to weak antilocalization in quantum transport property. In addition, this phase is a 2D topological insulator state when SOC is included. Our work provides a new 2D material platform to explore new topological emergent fermions and their interesting effects, which is of significant fundamental importance. Besides, the exotic physical properties and effects associated with these fermions are expected to make the materials promising candidates for electronic device applications. The strain‐induced metal–insulator quantum phase transition also makes these materials promising for making sensitive mechanical sensors.

## 4 Experimental Section

The first‐principles computations were based on the DFT, performed by implementing the projector‐augmented wave^59^ method in the Vienna ab initio simulation package.^60^ The generalized gradient approximation by Perdew, Burke, and Ernzerhof^61^ was used to extract the exchange‐correlation functional. A 20 Å vacuum layer was employed for all structures to avoid unphysical interaction between periodic images. Force and energy convergence criterion was set to 0.01 eV Å^−1^ and 10^−6^ eV, respectively. Energy cutoff was set to be 500 eV and the Brillouin zone was sampled using a 23 × 23 × 1 Γ‐centered k‐point mesh. Phonon spectra were calculated based on a 4 × 4 × 1 supercell by the finite displacement method using the PHONOPY package.^62^ For Na_2_O and K_2_O, which are the focus of the work, the effect of SOC is negligible, and hence the results without SOC are used for the discussion unless specified (see the Supporting Information).

## Conflict of Interest

The authors declare no conflict of interest.

## Supporting information

Supporting Information
