# Supplementary material for: Tunable Topological Energy Bands in 2D Dialkali‐Metal Monoxides
Source: Adv Sci (Weinh). 2020 Jan 7;7(4):1901939. doi: 10.1002/advs.201901939 (PMC7029633; doi:10.1002/advs.201901939)
Supplement: Supplementary file 1 — Supporting Information [file ADVS-7-1901939-s001.pdf]

Copyright WILEY-VCH Verlag GmbH & Co. KGaA, 69469 Weinheim, Germany, 2019.

ADVANCED SCIENCE
Open Access

Supporting Information

for *Adv. Sci.,* DOI: 10.1002/advs.201901939

Tunable Topological Energy Bands in 2D Dialkali-Metal Monoxides

Chenqiang Hua, Si Li, Zhu-An Xu, Yi Zheng, Shengyuan A. Yang,* and Yunhao Lu**

Supporting Information

Tunable Topological Energy Bands in Two Dimensional Dialkali-Metal Monoxides

Chenqiang Hua, Si Li, Zhu-An Xu, Yi Zheng, Shengyuan A. Yang* and Yunhao Lu**

In our two Dimensional (2D) Dialkali-Metal Monoxides, alkali-metals (Na and K) have low atomic mass which are small or comparable to phosphorus. Since potassium is the heaviest element in our systems, we calculate the bands of K_2O with SOC taken into consideration. As shown in **Figure S1**, we find negligible effects of SOC on the electronic bands of K_2O, by comparing with the result without SOC.

For the energy band of ML K_2O, which is shown in Figure 2b of the main text, there is a very small gap of ~3 meV between the E’ and A_1’ bands. Such a minigap suggests that the K_2O system is actually near the phase transition point (critical point), and small changes in calculation parameters may close the minigap. Since the gap is negligibly small, we can approximately regard that the E’ and A_1’ bands are intersecting at the Γ point to form a triply-degenerate point without losing the generality of phase transition (**Figure S3**a).

Beyond the quantum phase transition study, it is noticeable that the monolayer dialkali-metal monoxides with the 2H-TMDC structure enjoy excellent flexibility. Due to the low stiffness constants of DMMOs, it is expected that they can be used as strain sensor like other nanomaterial-based sensors.[1] Naturally, due to this low stiffness

constants, quantum phase transition could be likely applied for different devices. Near pseudospin-1 phase, super Klein tunnelling may be used for perfect focusing lens (strain tuned tunnelling).[2] Within Double Weyl state region, strain manipulated transport device could be fabricated, even with the transition from weak localization to weak anti-localization. Besides, in (tilted) Dirac phase (under compressive strain), due to electron-hole pockets, large magnetoresistance may appear under magnetic field for some practical applications.[3] Furthermore, although DMMOs are expected to be reactive to the surrounding atmosphere, the sensitivity may be utilized for vapor detection, as demonstrated by air-sensitive black phosphorus.[4]

Here, we summarize the properties of Li_2O, Rb_2O and Cs_2O. Firstly, we show the band structures of Li_2O with phonon spectrum included in **Figure S8**. We find no significant difference in the electronic structure from K_2O (Na_2O), all showing three energy bands of E' and A_1' near the Fermi-energy. The phonon dispersion almost proves the dynamical stability but with some imaginary part which may due to the calculation method and size difference (SD) between anions and cations. Now we move to Rb_2O and it has similar SD and also cause some imaginary phonon modes as Li_2O. But for Cs_2O, more imaginary modes will appear due to the biggest size and electronegativity difference as manifested in **Figure S9**. If we take the atomic size from Na to K as the safe region in which there won't have any imaginary phonon modes, Cs (Rb) atom will 0.4 (0.15) Å larger than K atom empirically (Li atom is 0.35 Å smaller than Na atom). These size differences are well in agreement with the magnitude of imaginary modes (Cs > Rb > Li).

Noticeably that near the Fermi-energy there are three energy bands of E' and A_1' in all A_2O systems. Among them, the first band E' change the dispersion significantly

(may due to the size and electronegativity differences when increasing the size of cation from Li to Cs). Therefore, in Rb_2O and Cs_2O, there are some differences in this three bands, compared to A_2O (A= Li, Na, K). For these two compounds, *E*' and A_1' are repulsed to each other and separated at Γ point, and simultaneously exhibit a valley at K point. At the same time, the aforementioned size and electronegativity differences will cause different hybridization between anion-*p* and cation-*p* (-*s*) orbitals, then change the dispersion of *E*'. For example, in **Figure S10**, we depicted the projected density of states of Na_2O and Cs_2O for comparison. The obvious difference is the distribution of O-*p* orbital in conduction bands (CBs) region near E_F: in Cs_2O, no O-*p* contribution could be found in the top of CBs while it will exist even in high CB region in Na_2O ML. Meanwhile, in valence bands (VBs) region, the contribution of cation-*p* is much higher in Cs_2O. These different hybridization mainly originating from the electronegativity (and size) discrepancies, further change the band structures.

Besides, we try to give some discussions about the multilayer Na_2O in the sequence of traditional MoS_2 (2H and 3R stacking). Both bilayer (BL) and trilayer (TL) have been summarized in **Figure S11**. For 2H stacking, BL will go into D_{3d} point group while TL remains the D_{3h} as ML, and BL (TL) of 3R stacking is in C_{3v} point group. These multilayers (point groups) actually keep the key *E* and *A* irreducible representations at BZ center. The optimized lattice constant of 2H-BL is 3.58 Å (3.62 Å for 2H-TL), which is larger than 3.49 Å of ML. The enlarged lattice constant may make some differences (like phase transition as ML). Exactly for 2H-BL, it breaks the original *E* representation and go into semiconductor phase may due to enlarged structure (and band repulsion), which indicates the interlayer interaction is not negligible. One have note that the top of VB is actually not 4-fold degenerate but in 2-

fold degeneracy with E representation (see insets of **Figure S11**). When it increases up to 2H-TL, it seems that bands will go back to ML situation (double Weyl) since they are in the same point group. However, like BL system, the interlayer interaction also favors the reconstruction of E representation and thus having phase transition to semiconductor phase as well. But for 3R-BL (TL), the E representation remains the original combination (CB and VB) so that it still in double Weyl phase. The survival of double Weyl phase may could be contributed to the small lattice change or the small interlayer interaction.

For the cohesive energy calculation, we using the definition as follows:

$$E_c = E_{A_2O} - (E_A \times 2 - E_O),$$

where E_{A_2O} is the energy of ML DMMO, E_A and E_O represent the energy of isolated alkali metal atom and oxygen atom, respectively. We summarize the cohesive energy of some 2D systems in **Table S1**. The values of cohesive energy of Na_2O and K_2O are actually large and even larger than some experimentally synthesized 2D materials.

Table S1. Cohesive energy of some 2D systems.

2D systmes	Cohesive energy (eV/f.u.)	Synthesized or not yet?
Te-ML	-7.34	yes
2H-MoS2	-15.06	yes
black P-ML	-14.27	yes

buckled Bi-ML	-5.48	yes
2H-Na2O-ML	-8.02	not yet
2H-K2O-ML	-7.36	not yet

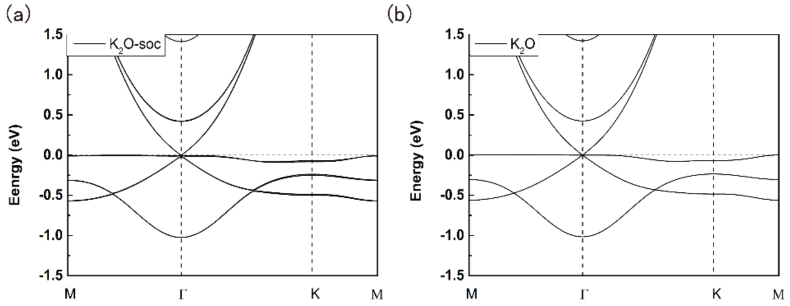

Figure S1. (a) and (b) The bands of ML K_2O with and without SOC taken into consideration.

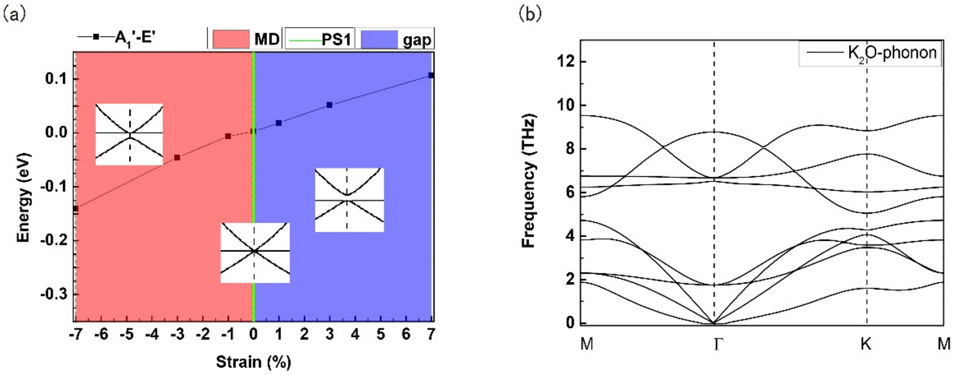

Figure S2. (a) The strain tunable phase diagram of ML K_2O. The line indicates the energy difference between the A_1' and E' irreducible representations. (b) Phonon spectrum of ML K_2O, showing no imaginary mode in the whole Brillouin zone.

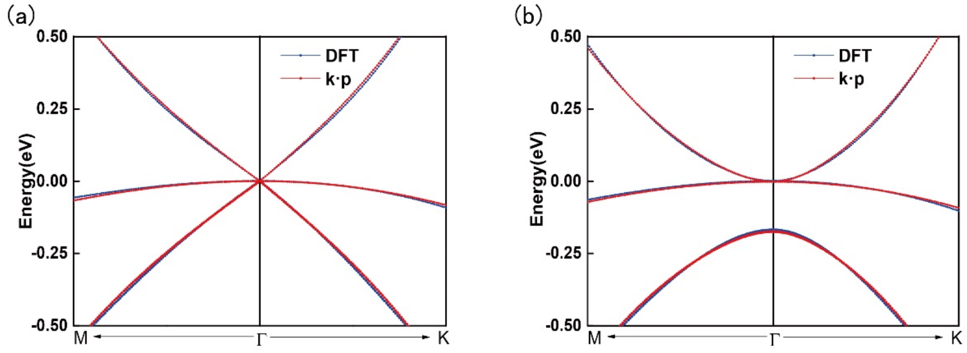

Figure S3. (a) - (b) are the fitting results of ***k·p*** model to the energy bands of the pseudospin-1 fermion state and the double Weyl (massive Dirac) state the in ML K_2O and ML Na_2O, respectively. The ***k·p*** fitting is represented by the red lines, while the DFT results are the blue lines.

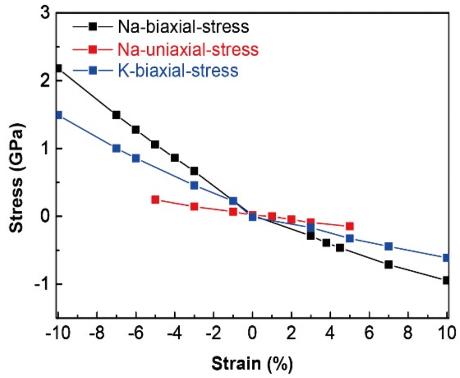

Figure S4. Strain-stress relationship for monolayer dialkali-metal monoxides. Most of the strain conditions are experimental feasible.

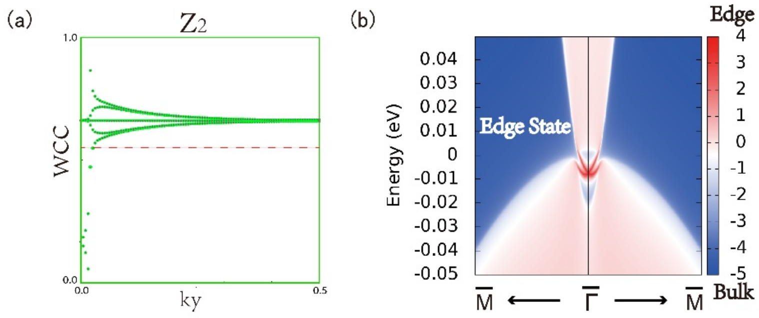

Figure S5. (a) Wannier charge center evolution of uniaxial strained ML Na_2O, when SOC is taken into account. The result indicates that Z_2 is one and the system is a non-trivial strong TI. (b) Topological edge state (red solid lines) of the strong TI state. The result is plotted by projected the bulk bands along the zigzag direction.

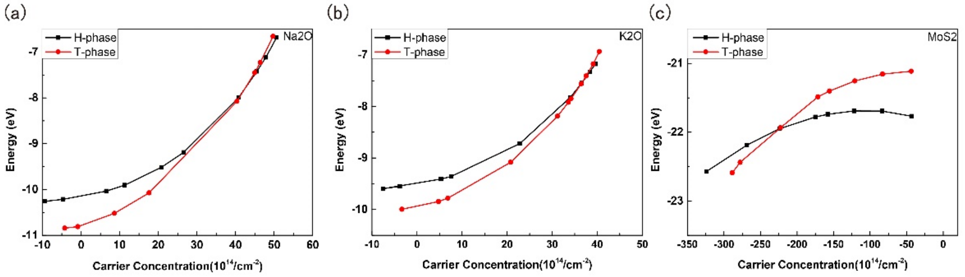

Figure S6. (a) - (c) are the energy evolutions of 1T and 2H phases in ML Na_2O, ML K_2O and MoS_2 three monolayer systems (primitive cell is used), respectively, as a function of charge carrier doping. The results suggest that ~40×10^{14} cm^{-2} hole doping concentration is necessary to reverse the energy difference between 1T and 2H phases, while high electron doping is needed for ML MoS_2.

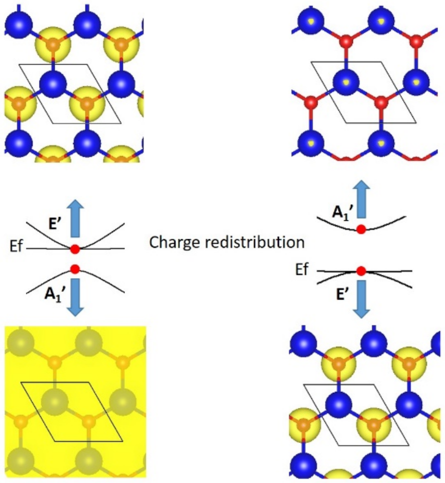

Figure S7. Real space charge distribution of the conduction band minimum and the valence band maximum of ML K_2O at Γ point. It is distinctive that charge redistribute drastically after the topological phase transition when the A_1' and E' bands reverse relative energy position.

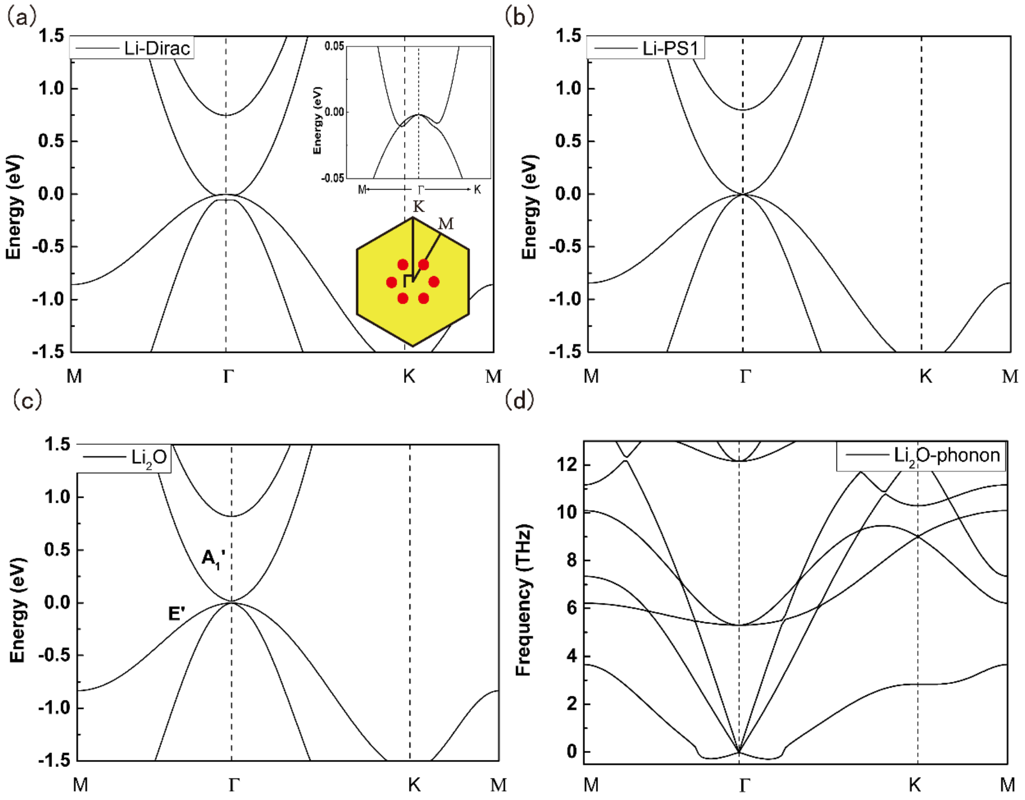

Figure S8. Band structures of ML Li_2O under biaxial strain of (a) -1%, (b) -0.3% and (c) 0%. Inset of (a) show the emergence of six symmetry protected single Dirac points (red dots) along Γ-M paths. (d) Phonon spectrum of ML Li_2O, showing no special imaginary mode in the whole Brillouin zone except the Γ point.

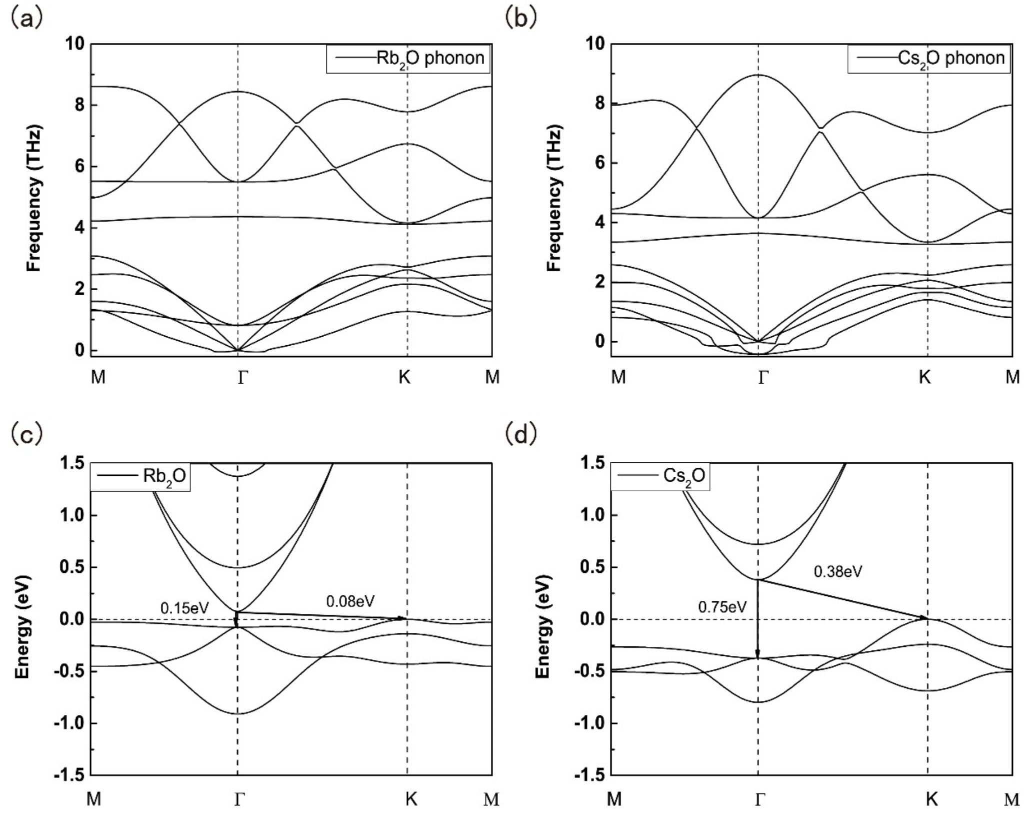

Figure S9. Phonon spectra of (a) ML Rb_2O and (b) ML Cs_2O with band structures of (c) ML Rb_2O and (d) ML Cs_2O.

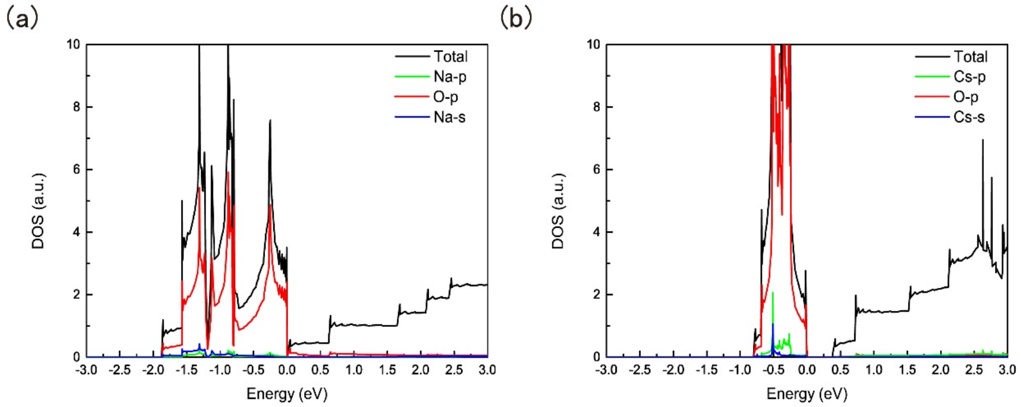

Figure S10. Projected density of states of (a) ML Na_2O and (b) ML Cs_2O.

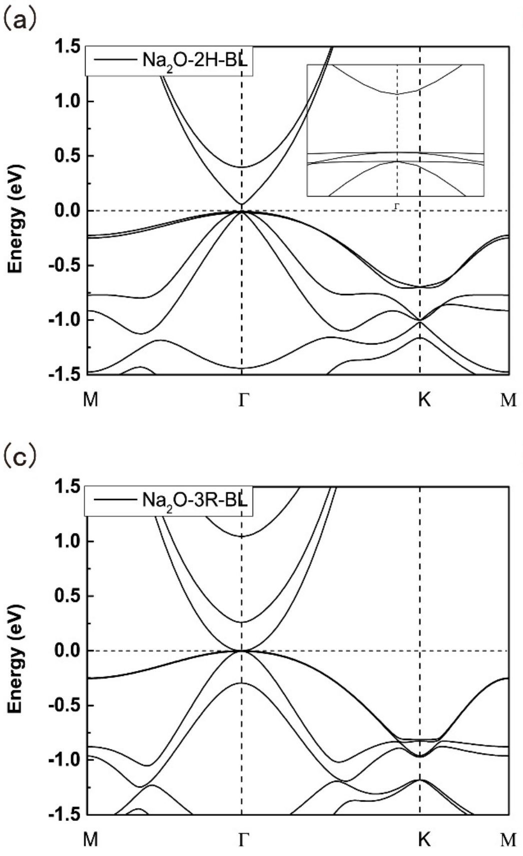

Figure S11. Band structures of BL and TL Na_2O. (a) and (b) are in 2H-stacking while (c) and (d) are stacked in ABC-3R sequence. Insets give the bands (VBs mainly) in representation E for 2H stacking Na_2O.

[1] C. C. Mayorga-Martinez, Z. Sofer, M. Pumera, *Angew. Chemie Int. Ed.* **2015**, *54*, 14317.

[2] D. F. Urban, D. Bercioux, M. Wimmer, W. Häusler, *Phys. Rev. B* **2011**, *84*, 115136.

[3] Y. Luo, N. J. Ghimire, M. Wartenbe, H. Choi, M. Neupane, R. D. McDonald, E. D. Bauer, J. Zhu, J. D. Thompson, F. Ronning, *Phys. Rev. B* **2015**, *92*, 205134.

[4] C. Yan, J. Wang, W. Kang, M. Cui, X. Wang, C. Y. Foo, K. J. Chee, P. S. Lee, *Adv. Mater.* **2014**, *26*, 2022.
